# Cystic Lymphangioma Over the Lower Limb: A Case Report With a Literature Review

**DOI:** 10.7759/cureus.53259

**Published:** 2024-01-30

**Authors:** Sagarika S Bhole, Mohd Yunus Shah, Zansher Nazar

**Affiliations:** 1 Surgery, NKP Salve Institute of Medical Sciences and Research Centre, Nagpur, IND

**Keywords:** lymphangioma, lower limb, mri, histology and histopathology, congenital

## Abstract

Cystic lymphangioma (CL) is a rare congenital deformity that almost exclusively affects children. Among the incidences, the majority occur in the head and neck area. Adults who develop these lesions likely develop them as a result of trauma. Here, we present the case of an adult female with a CL in the right calf region. Over time, it continued to grow to a noticeable size. Surgical resection was a good management strategy for the patient. We conducted a literature survey in light of scarce reports depicting CL in the limbic area.

## Introduction

Based on histology, lymphangiomas are categorized as capillary, cavernous, or cystic. In 1913, Koch originally characterized cystic lymphangioma (CL) as a benign tumor with a vascular abnormality. It usually occurs as a result of a lymphatic system developmental defect that causes the lymphatic tissues to sequester. CL exhibits expansive lymphatic compartments containing collagen and lymphocytes [1,2]. Adult lymphangiomas are thought to arise from the delayed growth of lymphatic channels. These tumors are special in that they can grow larger and develop lymph along with the cystic cavities. It is an uncommon tumor that is frequently seen in children and the neck and head areas [3]. CL at other places is uncommon and occasionally presents a diagnostic conundrum. Here, we present the case of a 25-year-old female with a CL across her right leg.

## Case presentation

Clinical presentation

A 25-year-old female presented with a history of swelling over her right lower limb in the calf region since birth. The swelling was static and painless without causing difficulty in limb movements. There was no past medical history of hypertension, diabetes mellitus, tuberculosis, or asthma. Moreover, there was no previous history of trauma, radiation exposure, or infection.

Clinical finding

**Figure 1:**
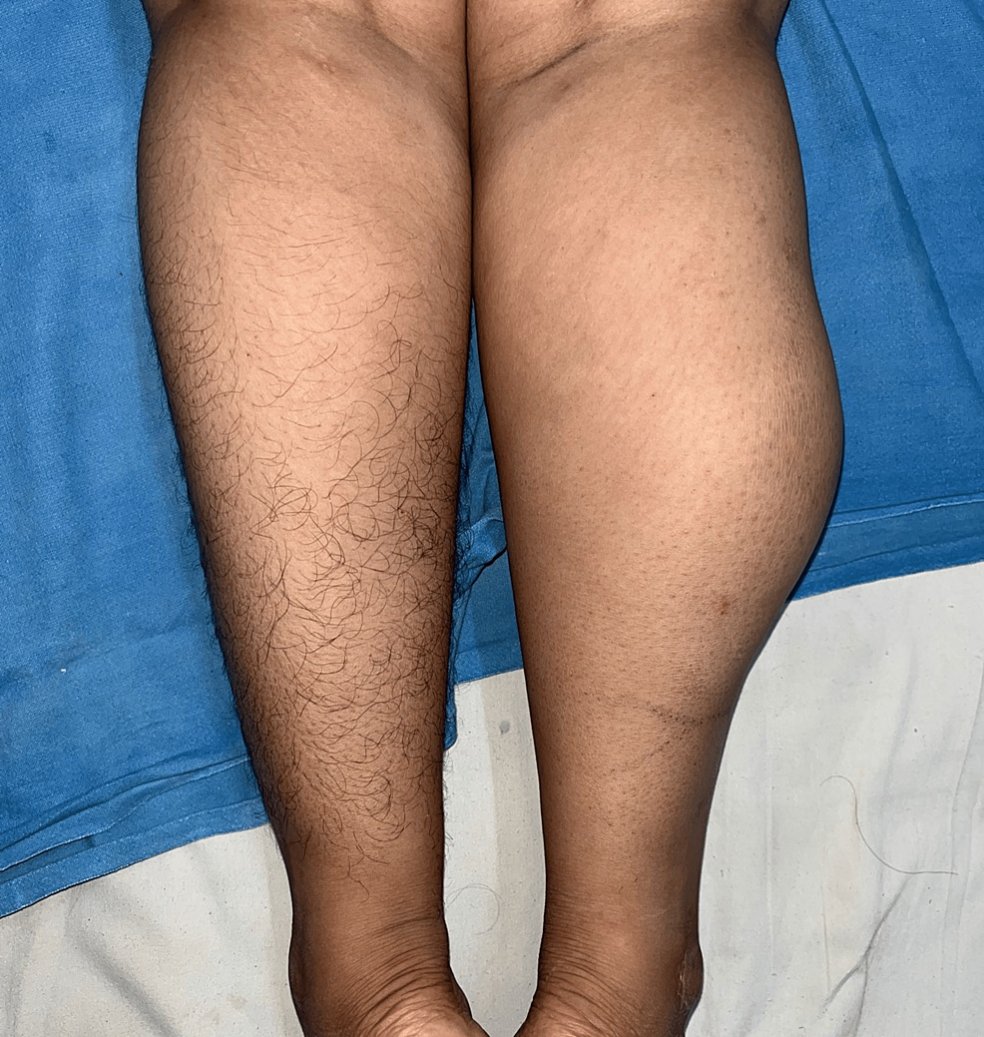
Preoperative view (posterior).

**Figure 2:**
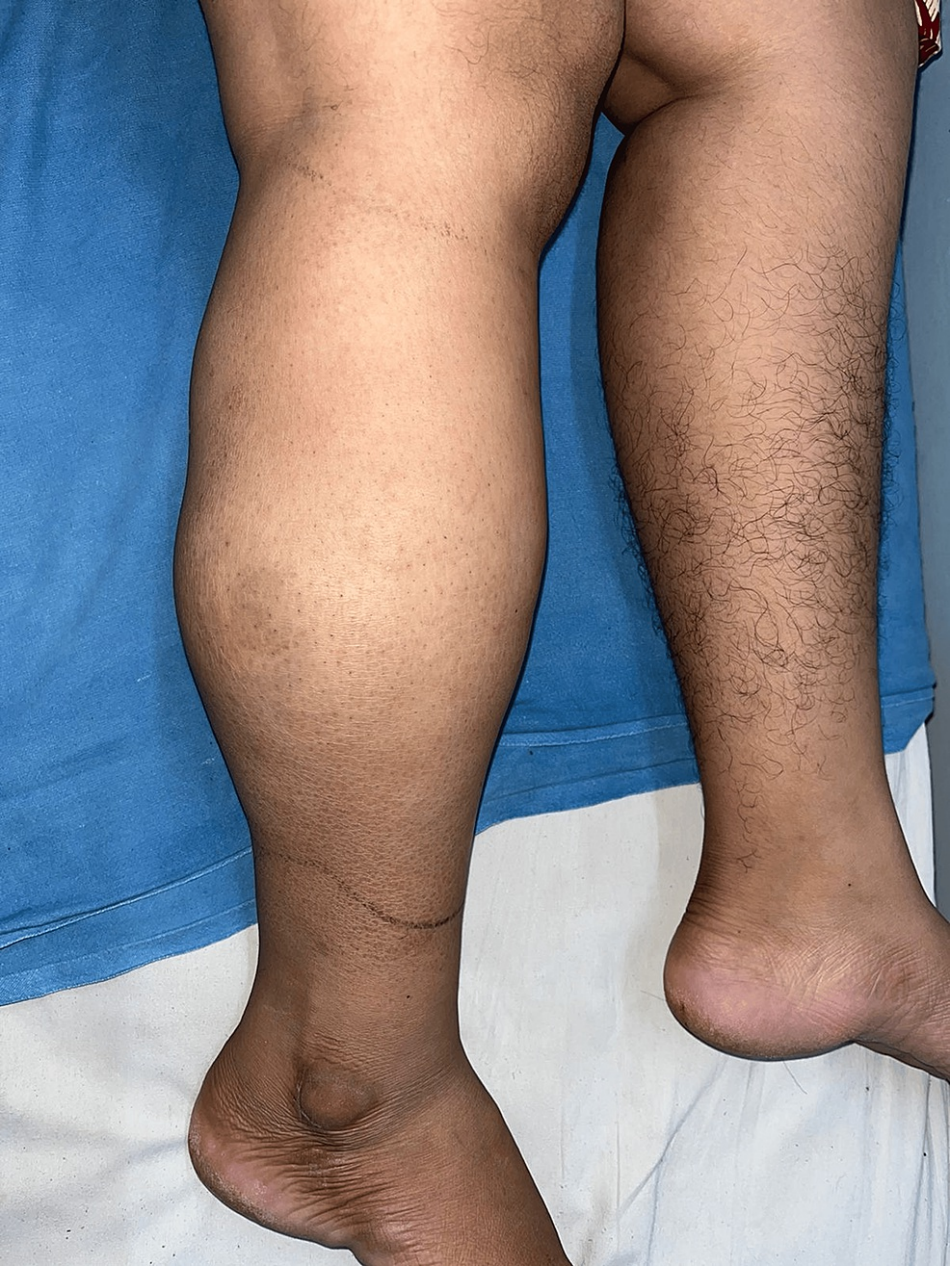
Preoperative view (lateral).

On clinical examination, a swelling of 16 x 12 cm was seen involving the lateral and posterior compartments of the right leg. A few bluish discoloration spots were noted over the skin of the swelling. The swelling was oval with firm consistency and restricted mobility (Figures 1, 2). It was non-tender with no local rise in temperature. There was no restriction of movement or change in the size of the swelling on the dorsi/plantarflexion of the foot.

Diagnostic assessment

Ultrasonography demonstrated an ill-defined soft tissue lesion containing multiple cystic areas in the subcutaneous plane suggestive of lymphatic malformation.

Right lower limb arteriovenous Doppler noted multiple interconnected tubulocystic pockets of fluid in the subcutaneous plane below the knee up to the mid-calf in the anterior and posterolateral aspect of the leg, with focal areas of vascular necrosis suggestive of lymphatic malformation with focal areas of vascular malformation.

**Figure 3:**
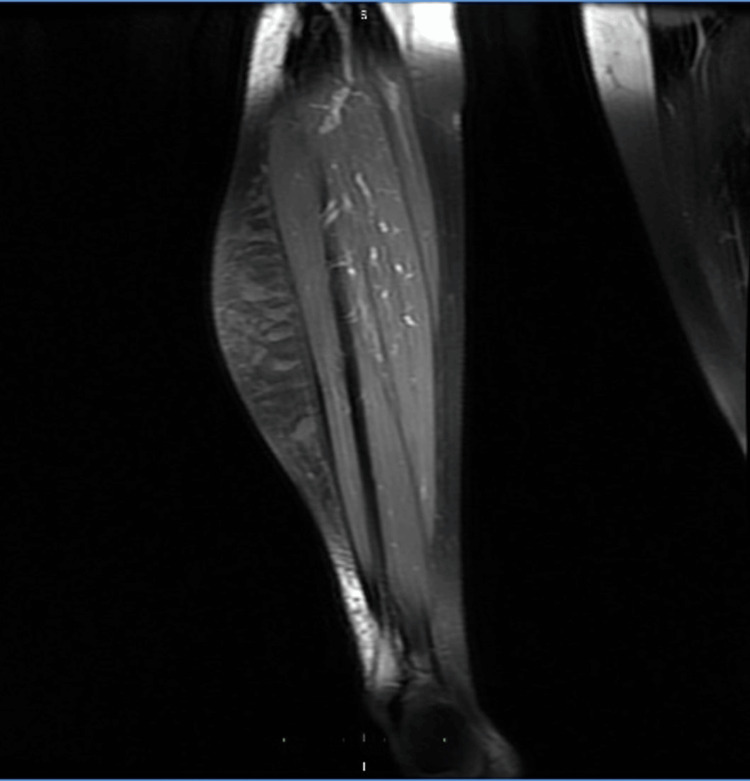
Coronal T1 view showing the lesion in the intermuscular plane which appears as a low signal on T1-weighted imaging and shows homogenous suppression. The fat plane is maintained with the adjacent muscles.

**Figure 4:**
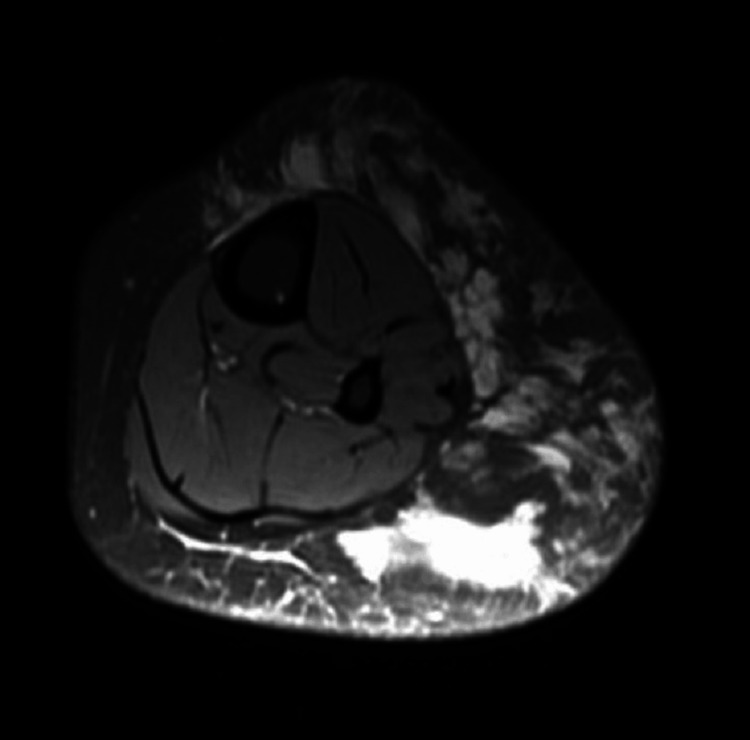
Fat-saturated view showing a multiloculated high-signal intensity lesion in the intermuscular plane between the soleus and lateral head of the gastrocnemius muscles. Multiple thin internal septations are seen predominantly in the superior aspect.

Magnetic resonance imaging (MRI) of the right leg demonstrated a large ill-defined multiloculated cystic non-enhancing lesion in the subcutaneous plane in the anterior and posterolateral aspect of the right leg from the knee to the ankle, with maintained fat planes suggestive of lymphatic malformation (Figures 3, 4).

Intervention

**Figure 5:**
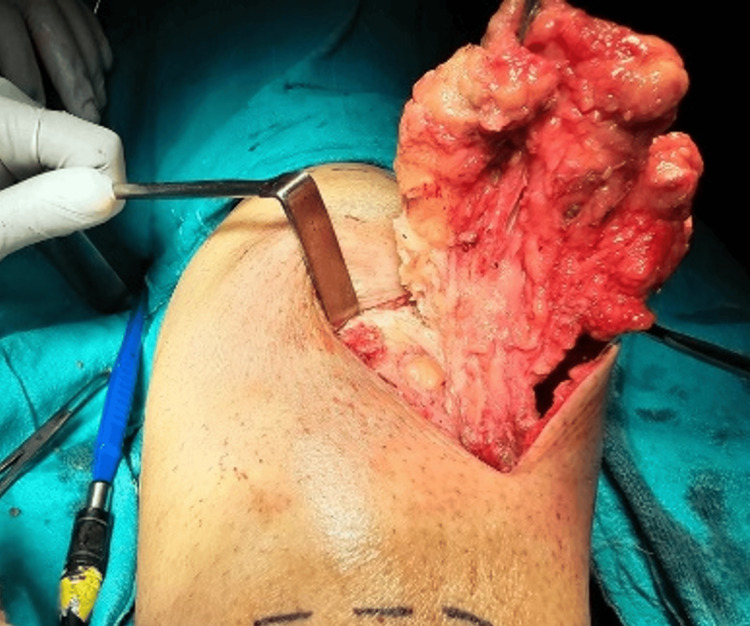
Excision of the swelling.

**Figure 6:**
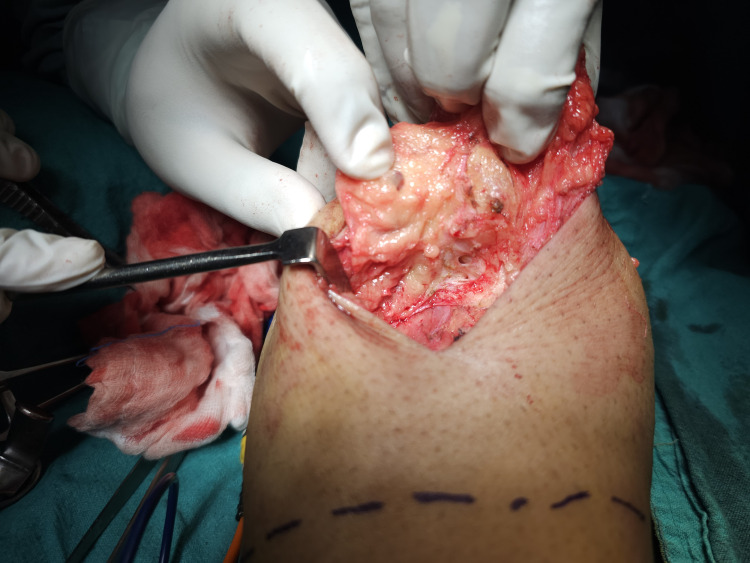
Lesion showing cystic cavities filled with lymph.

**Figure 7:**
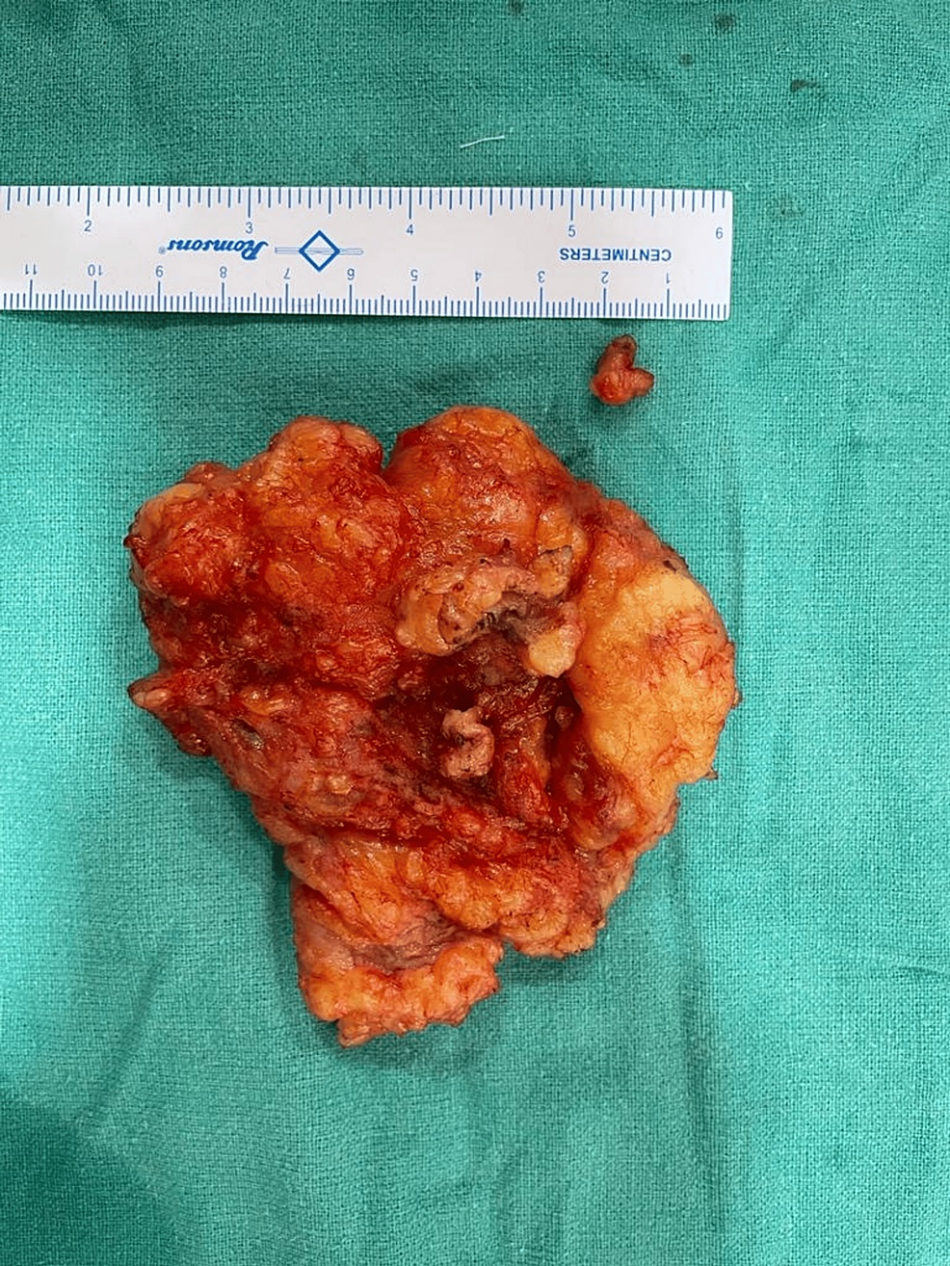
Excised mass measuring 12 x 10 cm.

The patient was posted for surgical exploration and excision of swelling. Intraoperatively, a swelling measuring approximately 12 x 10 cm with multiple lymph-filled cavities was noted. It was superficial to deep fascia. Complete excision was achieved (Figures 5-7), and a negative suction drain was placed.

Follow-up and outcomes

**Figure 8:**
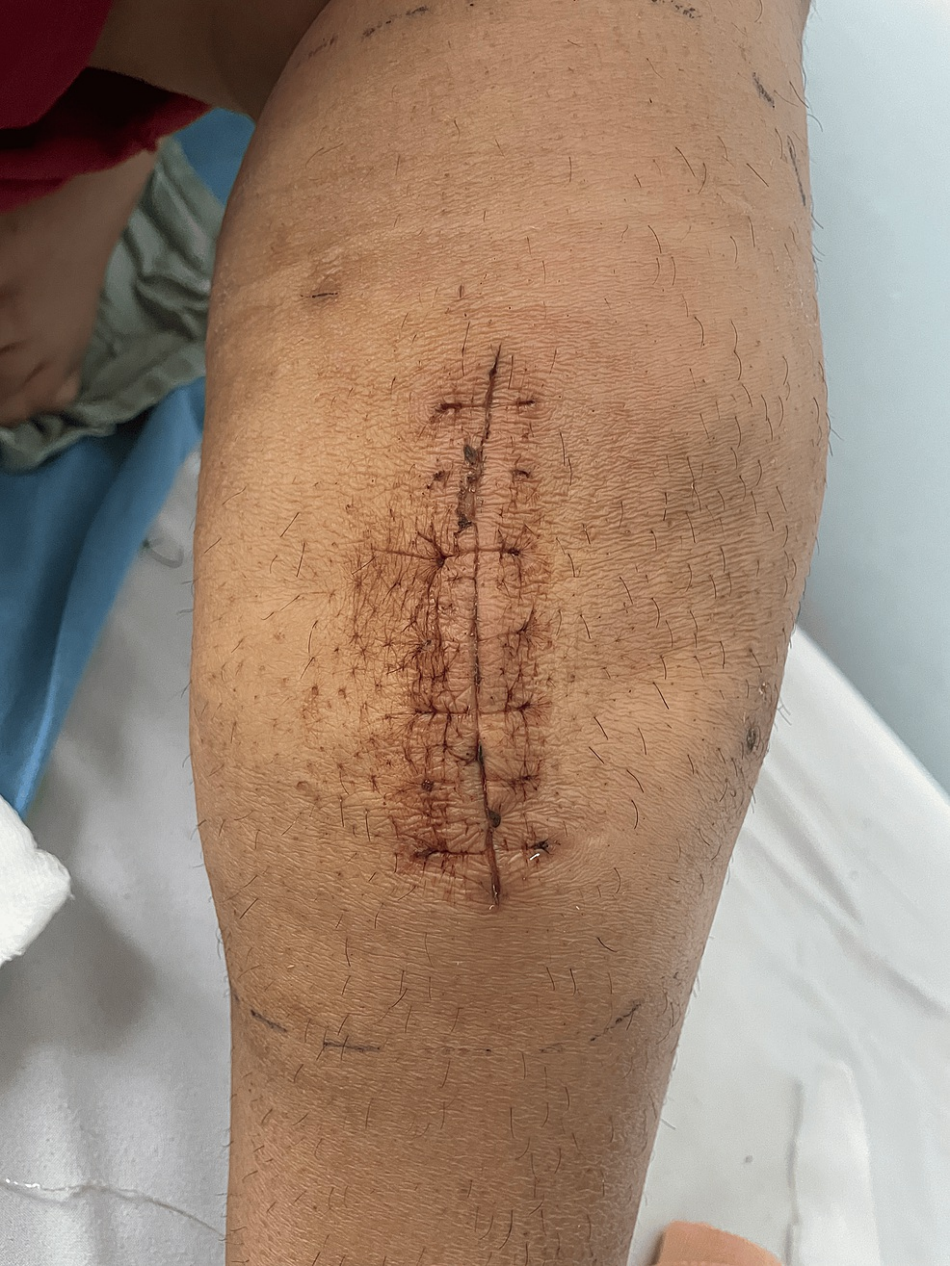
Postoperative healing after 10 days.

The postoperative course was uneventful and the patient was discharged on postoperative day 10 after drain removal (Figure 8).

Histopathological assessment

**Figure 9:**
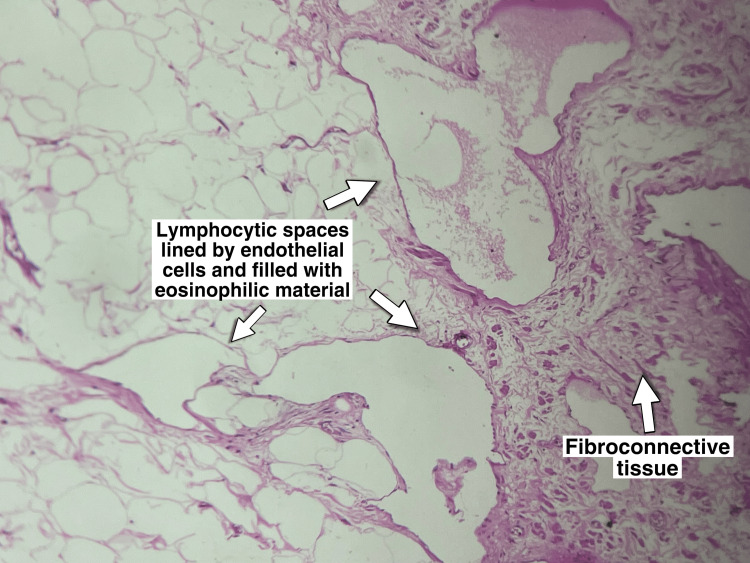
Lymphocystic spaces lined by endothelial cells and filled with eosinophilic material.

**Figure 10:**
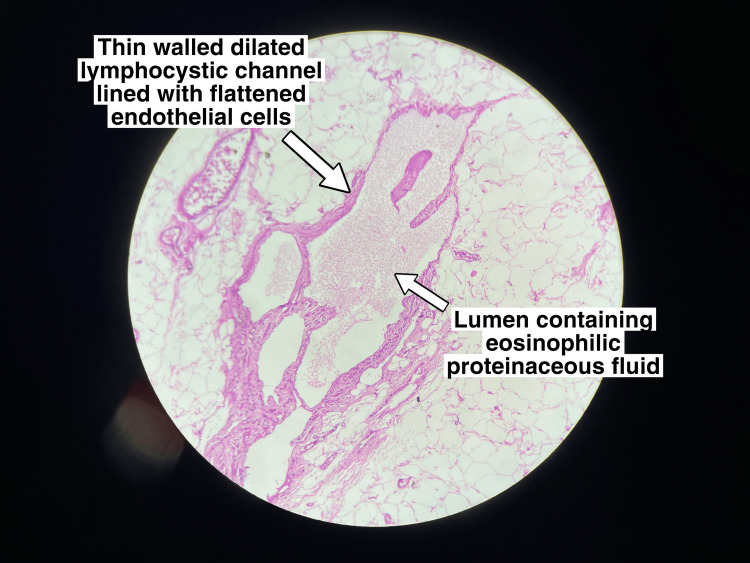
Thin-walled dilated lymphocystic channel lined with flattened endothelial cells and lumen consisting of eosinophilic proteinaceous fluid.

Histopathology reported irregular fragments of tissue composed of cystically dilated lymphatic spaces lined by flattened endothelial cells suggestive of CL (Figures 9, 10).

## Discussion

Congenital lymphatic system abnormalities that cause lymphatic tissues to sequester during infancy cause CLs, which are frequently observed in the head and neck areas [1,2].

We examined earlier research on the occurrence of CL in limbs because our patient’s lower limb presented with a CL at an uncommon location. The terms “Cystic hygroma,” “Lymphangioma,” “Cystic Lymphangioma,” and “Limbs” were used to search PubMed, Scopus, Web of Science, and Google Scholar databases. No additional filters were used for language, study design type, or chronology. We reviewed all available studies that included a thorough case presentation and lesion management.

We examined 10 articles that explored the presentation of CL on the limbs (Table 1). Despite the age range of eight months to 62 years, females are more frequently affected than males. CL affecting the limbs may present as painless swelling over the affected area. Most examinations in females showed soft, non-tender lumps with a doughy consistency upon palpation.

**Table 1:** Characteristics of studies assessing cystic lymphangioma of the limbs.

Authors	Type of study	Patient characteristics: age /gender	Limbic system involved	Site	Tumor size	Management	Follow-up
Douglas-Seidl et al. 2023 [4]	Case report	62/F	Lower	Right thigh	12 × 11 × 6.8 cm	Surgical excision	1 week
Zhu et al. 2023 [5]	Case report	42/F	Upper	Right upper forearm	6 × 1 cm	Radical surgical resection	7 months
Diarra et al. 2022 [6]	Case report	5/F	Upper	Right upper forearm	Not reported	Partial and wide surgical resection	Not reported
Yoshida et al. 2022 [7]	Case report	18/F	Upper	Right axilla	12 × 8 cm	Lymphaticovenular anastomosis + ethanol sclerotherapy	18 months
Cui et al. 2021 [8]	Case report	42/F	Upper	Right upper forearm	6 × 1 cm	Wide surgical resection	Not reported
Padmanaban et al, 2021 [9]	Case report	30/M	Lower	Right calf region	7.2 × 4.6 × 5.8 cm	Wide local excision	Not reported
Rahal et al. 2019 [10]	Case report	8 months/M	Upper	Right elbow	3 × 2 × 4 cm	Radical excision	6 months
Thakur 2010 [11]	Case report	7/M	Lower	Left upper and inner aspect of the thigh	5 × 4 cm	Surgical excision	Not reported
Hadj-Henni et al. 2008 [12]	Case report	21/F	Lower	Right lower limb	34 × 52 × 40 mm	Elastic compression by tights (stockings) (class 3)	11 months
Sinha et al. 2008 [13]	Case report	52/M	Lower	Upper part of the left thigh	20 × 30 × 30 cm	Wide surgical resection	Not reported

Typically, aspiration is used to identify the cystic lesion, primarily revealing straw-colored fluid [6-10]. Nevertheless, there is a significant chance of recurrence, and infection can result from repeated aspirations. Imaging plays the main role in the detection and characterization of cystic content either with ultrasound, computed tomography (CT), and/or MRI. On ultrasound investigation, the tumor appears as a simple or multiloculated cystic mass with internal septations. On CT, tumor masses show densitometric characteristics of the fluid type, regular margins, and only a peripheral contrast enhancement. Although less available than CT, MRI allows a clear evaluation of the lesion morphology and structure, showing vessel-like internal septa, wall thickness, and fluid content, excluding the presence of mucoid, adipose, or solid components. Hence, imaging techniques can be utilized to preoperatively evaluate the extent of infiltration and the tumor’s relationship to significant structures in complex anatomical locations such as the neck [14]. However, a histological analysis of the excised surgical specimen confirms the diagnosis.

The histological features under the microscope reveal flattened endothelial cells and dilated lymphatic channels. Under a microscope, the material reveals a soft, multiloculated cystic mass that contains lymphatic fluid. The mass may occasionally contain fluid that is serous or serosanguinous [15].

There is no record of CLs transforming malignantly. CLs often have a good prognosis and are benign [16].

There is still no agreement on how to manage CLs. The management techniques mentioned in the literature include aspiration, sclerotherapy, cryotherapy, electrocautery, radiation, laser, ligation, and excision. Nonetheless, care be tailored to the specific lesion’s size, anatomic location, and consequences [6-14].

The cornerstone of lesion management in symptomatic cases is surgery. To ensure the safe removal of the entire tumor, care should be taken not to decompress the cystic lesion during removal. Although other sclerosing drugs, including bleomycin (0.3-0.6 mg/kg) [17,18] and 0.2 mg OK-32(a lyophilized mixture of group A *Streptococcus pyogenes* and benzylpenicillin, marketed as Picibanil) [19], have been used in injection therapy, their usefulness in complex patients is debatable. Sclerotherapy and lymphaticovenular anastomosis combined have recently been proposed as a comparatively less invasive technique that closes the dead space without aggravating or causing lymphedema [7].

## Conclusions

Adults with CL are uncommon, especially when it involves the lower limb. We provide an account of the infrequently reported cases presenting in the calf region. Adults with these tumors may have late appearances that are challenging to diagnose but are easily treated with surgery and meticulous dissection. Because decompressing the cyst during excision increases the risk of partial excision and consequent recurrence, caution should be exercised.
